# Pinpointing the integration of artificial intelligence in liver cancer immune microenvironment

**DOI:** 10.3389/fimmu.2024.1520398

**Published:** 2024-12-20

**Authors:** Ihtisham Bukhari, Mengxue Li, Guangyuan Li, Jixuan Xu, Pengyuan Zheng, Xiufeng Chu

**Affiliations:** ^1^ Department of Oncology, The Fifth Affiliated Hospital of Zhengzhou University, Zhengzhou, China; ^2^ Marshall B. J. Medical Research Center, Zhengzhou University, Zhengzhou, Henan, China; ^3^ Department of Gastrointestinal & Thyroid Surgery, The Fifth Affiliated Hospital of Zhengzhou University, Zhengzhou, China

**Keywords:** liver cancer, immune microenvironment, artificial intelligence, machine learning, ScRNA-seq

## Abstract

Liver cancer remains one of the most formidable challenges in modern medicine, characterized by its high incidence and mortality rate. Emerging evidence underscores the critical roles of the immune microenvironment in tumor initiation, development, prognosis, and therapeutic responsiveness. However, the composition of the immune microenvironment of liver cancer (LC-IME) and its association with clinicopathological significance remain unelucidated. In this review, we present the recent developments related to the use of artificial intelligence (AI) for studying the immune microenvironment of liver cancer, focusing on the deciphering of complex high-throughput data. Additionally, we discussed the current challenges of data harmonization and algorithm interpretability for studying LC-IME.

## 1 Introduction

Liver cancer poses huge health challenges due to escalating global incidence, notably in transitional regions like East and Southeast Asia. It currently ranks 6^th^ in cancer incidence and 3^rd^ in mortality, surpassed only by lung and colorectal cancers (1). Surgery provides relatively satisfactory outcomes when detected at an early stage, liver transplantation at early-stage liver cancer patients achieved a 5-year survival of about 70-80.0%. Surgical resection or tumor ablation can reach a 5-year survival rate of 50% to 70% (2–5). For patients with locally advanced liver cancer, Trans-arterial Chemoembolization (TACE), either in combination with other treatments or as a standalone therapy, yields a 5-year survival rate of 20% to 40% (4, 6).

Systemic therapy has witnessed significant breakthroughs in targeted therapy and immune therapy in the past two decades, which have not only improved survival in advanced patients but also made some of them suitable for surgical removal. Even so, liver cancer remains one of the worst-prognosed diseases due to late diagnosis, drug resistance, and frequent recurrence and metastasis (7). The chances of survival of the patients with liver cancer at late stage are low due to the lack of effective drugs, meaning that patients typically live for only 6 to 20 months after diagnosis (8). This underscores the urgent need for effective treatments (9).

Liver cancer has several subtypes, including hepatocellular carcinoma (HCC), bile-duct cancer, hepatoblastoma, and various liver sarcomas and carcinomas. Among them, HCC is the most common worldwide, whereas, in some Asian countries, bile-duct cancer is more common than HCC. This regional variation may result from different risk factors, such as hepatitis B virus, hepatitis C virus, fungi, aflatoxin, alcohol, poor diet, and parasitic flatworm (10, 11). It is still unclear that why some people can live with liver disease for many years, whereas others develop fatal cancer. Increasing evidence suggests the alterations of the liver immune microenvironment play a key role during cancer transformation and drug resistance. However, the heterogeneity and intricate molecular dynamics impede a deep understanding of the immune microenvironment of liver cancer.

In this review, we first provide a brief overview of AI and describe its common applications in cancer research. We also illustrated the immunological characteristics of the liver and its pathological alterations during cancer development. Subsequently, we explored the latest applications of AI and current challenges within the context of LC-IME.

## 2 Applications of AI in cancer research

### 2.1 AI and machine learning

AI technology involves the development of systems capable of executing tasks typically requiring human intelligence, such as reasoning, learning, and problem-solving. It is designed to replicate cognitive processes like perception, language processing, and decision-making, these systems draw from a diverse range of disciplines, including computer science, mathematics, psychology, and linguistics. AI technology has penetrated all aspects of human activities (12–15). In the cancer research field, AI is characterized by the use of machine learning and deep learning algorithms (16), which are important in processing and analyzing large-scale datasets (17–19).

**Figure 1:**
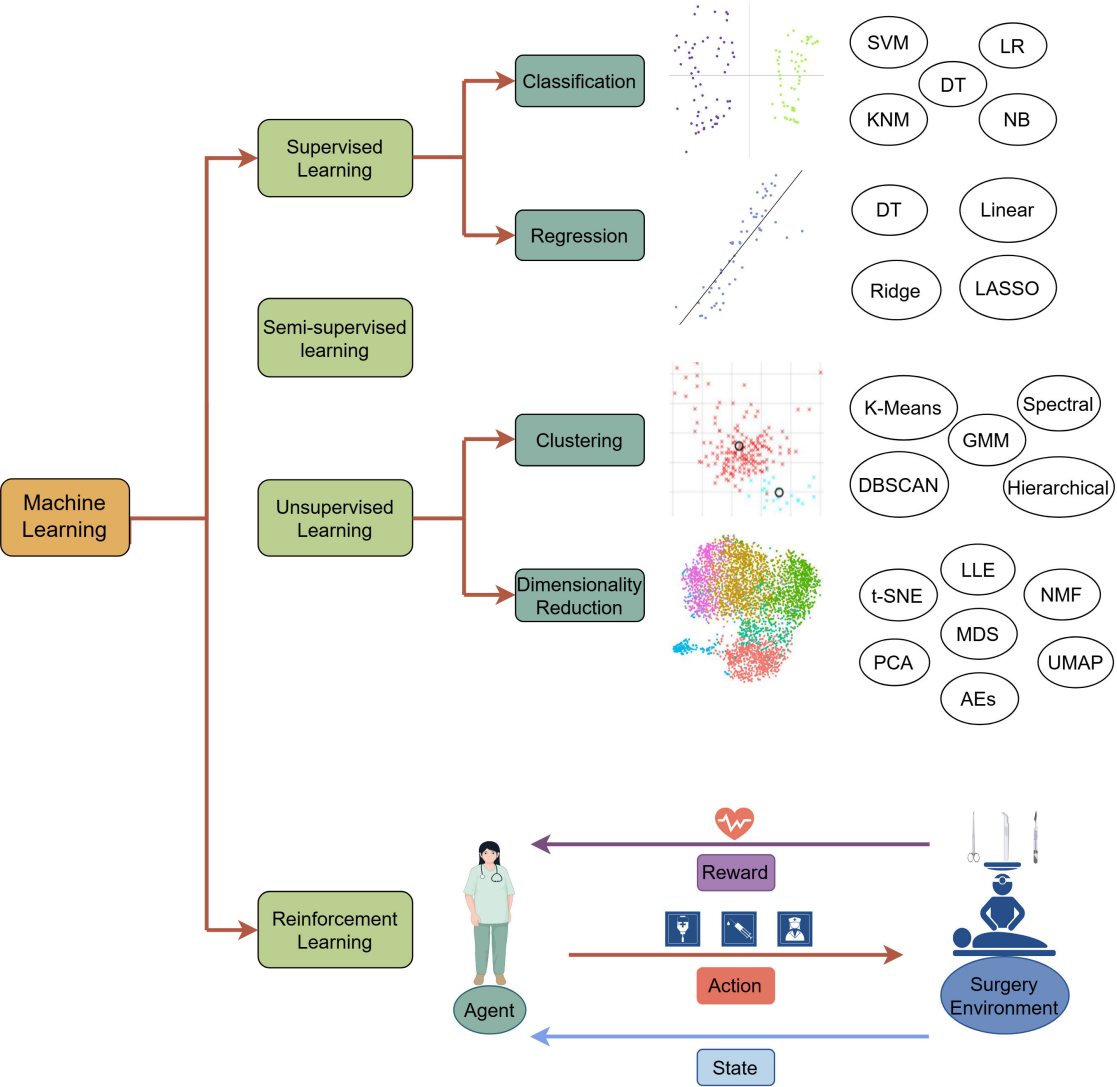
The overview of machine learning paradigms. Supervised learning: Trains models on a labeled dataset, where the training dataset consists of input data and corresponding output labels, allowing the model to be able to make accurate predictions on classification and regression tasks. Semi-supervised learning: Trains the model with a small amount of labeled data and applies the model trained to annotate unlabeled data. Unsupervised learning: Discovers hidden patterns, structures, or subgroups in the unlabeled data through clustering and dimensionality reduction. It uses datasets without clear notice of the dependent (response) variable. Unsupervised means that the machine or computer should learn patterns from the data without referring to any specific response. Unsupervised learning aims to explore the data structure and generate a hypothesis rather than to test any hypothesis by statistical methods or to construct prediction or classification models on the basis of a set of conditions and a specified response. Algorithms for unsupervised learning can be subdivided into two categories: (1) clustering algorithms and (2) dimensionality reduction. Reinforcement learning: Identifies a sequence of actions to increase the probability of achieving a predetermined goal. A RL problem is solved through a trial-and-error learning process. A RL agent interacts with an environment to maximize the cumulative reward resulting from its actions. Generally, RL problems are modeled and solved using a Markov Decision Process (MDP), guided by Bellman’s equation. There are four components: (1) a state that represents the environment at each time step; (2) an action the agent takes at each time step that influences the next state; (3) a transition probability that provides an estimate for reaching different subsequent states, which reflects the environment in which an agent interacts; and (4) a reward function, which is the observed feedback given a state-action pair. LR, Logistic Regression; DT, Decision Tree; NB, Naïve Bayes; SVM, Support Vector Machine; NN, K-nearest Neighbor; Ridge, Ridge Regression; Linear, Linear Regression; LASSO, Least Absolute Shrinkage and Selection Operator; GMM, Gaussian Mixture Model; DBSCAN, Density-Based Spatial Clustering of Applications with Noise; PCA, Principal Component Analysis; MDS, Multidimensional Scaling; NMF, Non-negative Matrix Factorization; LLE, Locally Linear embedding; t-SNE, t-Distributed Stochastic Neighbor Embedding Algorithm; UMAP, Uniform Manifold Approximation and Projection; AEs, Autoencoders.

Machine learning (ML), an integral part of artificial intelligence, encapsulates the autonomous identification of patterns and formulations within vast datasets (20). By discerning and extracting significant features from the data, ML can make accurate predictions and decisions .It fundamentally extracts patterns and rules from the data and apply them to new data. The workflow of ML comprises the acquisition, pre-processing, feature extraction of the data, model training, and evaluation optimization application of the obtained model. During model training, parameters are adjusted to minimize the discrepancy between predicted and actual outcomes, known as ‘error’ or ‘loss,’ which is quantified to direct the optimization process towards enhanced accuracy. Based on the model training approaches, there are four different types of ML: supervised (21), unsupervised (22, 23), semi-supervised (24), and reinforcement learning (25) (**Figure 1**).

**Figure 2:**
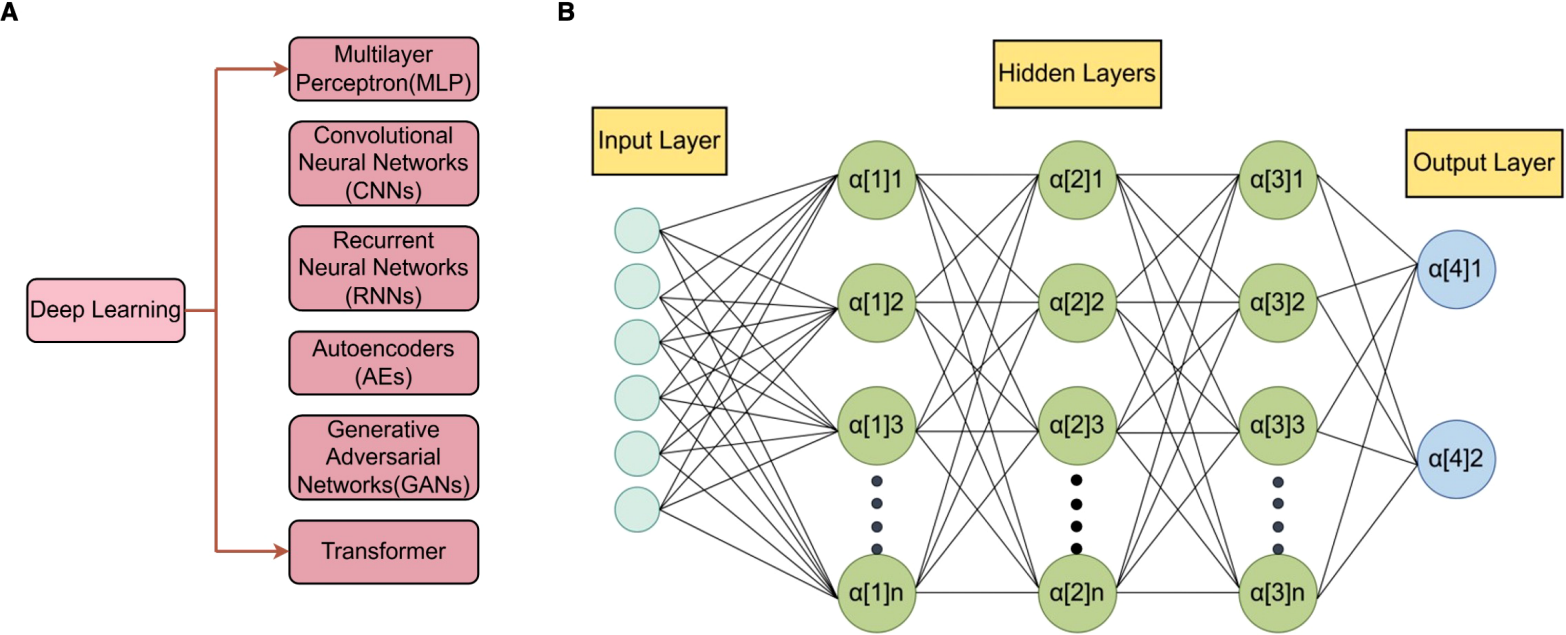
The overview of deep learning paradigms. **(A)**, Deep learning is a subfield of machine learning. It employs artificial neural networks for representation learning from massive amounts of data. A deep neural network consists of an input layer, multiple hidden layers, and an output layer, each of which receives the output of the previous layer of neurons as input and performs nonlinear transformation processing, thereby gradually transforming the raw data into meaningful feature representations. **(B)**, The composition of multilayer perceptron (MLP) is shown as an example of deep learning. The input layer receives data, where each neuron corresponds to a feature of the input data. The hidden layers perform computations and transformations on the input data through weighted sums and non-linear activation functions. These processed signals are then conveyed to the output layer, which generates the final output of the network.

Deep learning (DL), a subfield of machine learning, employs artificial neural networks (26) to represent important information from massive amounts of data. DL comprises an input layer, multiple hidden layers, and an output layer, each of which receives the output of the previous layer as input and performs nonlinear transformations that progressively distill raw data into meaningful feature abstractions. There are several popular DL architectures: multilayer perceptron (MLP), convolutional neural networks (CNNs), recurrent neural networks (RNNs), auto-encoders (AEs), generative adversarial networks (GANs) and transformer (**Figure 2**). These architectures can be used according to the specificity of the data.

### 2.2 Application of AI in cancer research

Early cancer detection: By facilitating cancer detection at the precancer stage, AI allows for early interventions that significantly prolong the overall survival time of the patients. For example, Klein et al. used a blood-based multi-cancer early detection (MCED) test and applied cell-free DNA sequencing, combined with machine learning, which predicted the origin of cancer signals with high specificity and accuracy in a variety of cancers (27). Similarly, Stark et al. constructed machine learning models using Gail model inputs and personal health data. These models exhibit strong performance in predicting breast cancer risk and can be used as non-invasive tools to increase early detection and prevention of breast cancer (28). Additionally, to develop a machine learning model to predict the risk of lymph node metastasis in renal carcinoma, Feng et al. filtered clinical features through LASSO and univariate and multivariate logistic regression analyses and then used statistically significant risk factors to build the XGB model. It could distinguish about 89% of LNM patients when the threshold probability was set to 54.6%, suggesting a promising application prospect in the clinic (29).

Machine learning and deep learning emerge as potent tools to identify biomarkers from intricate datasets (30). For example, Halner et al. established a random forest-based machine learning pipeline, “Decancer,” to analyze liquid biopsies. Decanter enhanced the sensitivity for detecting stage I cancer from 48% to 90% regardless of cancer type. Promisingly, DEcancer’s performance using a 14-43 protein panel is comparable to 1,000 original proteins (31). To identify metabolomic biomarkers for the diagnosis and prognosis of gastric cancer, Chen et al. used the LASSO regression algorithm to build a 10-metabolite GC diagnostic model, which is validated in an external test set with a sensitivity of 0.905. This model exhibited superior performance to traditional models that utilized clinical parameters and identified two distinct biomarker panels, enabling early diagnosis and prognosis of cancer (32). Tayob et al. developed the parametric empirical Bayes algorithm and the Bayesian screening algorithm to improve the early detection of cancer, which improved sensitivity to cancer biomarkers (33). Furthermore, Konstantinos et al. tested the miRNA expression profiles of Gastrointestinal stromal tumors (GISTs) and applied machine learning to identify the miRNAs associated with the risk of GIST development. They found that several miRNAs, with hsa-miR218-5p as the best, may strongly affect the prognosis of GISTs and can serve as predictors for their development (34). In short, the application of AI and machine learning in oncology clinics has improved diagnostic time and clinical outcomes in various cancers.

Medical imaging: Imaging is at the forefront of clinical care. The integration of AI into image interpretation helps radiologists streamline workflow and improve patient care (35). Within imaging, convolutional neural networks (CNNs) and Deep Learning (DL) are exceptionally useful in computer vision and enable machines to see and interpret visual data (36, 37). Al-Masni et al. developed the ROI-based Convolutional Neural Network “You Only Look Once (YOLO)” to accurately detect and classify the masses in mammograms. It achieves an overall accuracy of 96.33% in detecting the mass location and 85.52% in distinguishing between benign and malignant lesions (38). Zhao et al. built the deep-learning-based, fully automated lymph node detection and segmentation (auto-LNDS) model based on multiparametric magnetic resonance imaging (mpMRI). The auto-LNDS achieved a sensitivity, PPV, and FP/vol of 80.0%, 73.5%, and 8.6 in internal testing and 62.6%, 64.5%, and 8.2 in external testing, respectively, significantly better than the performance of junior radiologists, therefore holding great potential for facilitating N-staging in clinical practice (39). Jin et al. developed a CNN-based algorithm to Improve the accuracy in Optical Diagnosis of Colorectal Polyps. It increased the accuracy of novice endoscopists to 85.6% and significantly reduced the skill-level dependence of endoscopists and costs (40).

Pathological identification: As the gold standard for confirming cancer, pathological identification holds paramount significance in diagnosis, prognosis, and therapeutic strategies. However, the heterogeneity of tumors poses a big challenge to precise diagnosis (41, 42). AI has transformed the landscape of cancer pathology by empowering it with enhanced diagnostic accuracy and streamlined decision-making frameworks, leveraging sophisticated histology image analysis (43). For example, to achieve an AI-based pathological prediction of the origins of unknown cancers. Lu et al. build the Tumor Origin Assessment via Deep Learning (TOAD), a deep-learning-based algorithm that provides a differential diagnosis for the origin of the primary tumor based on routinely acquired histology slides (44). In addition, Lee et al. presented a graph deep neural-network model to analyze the whole-slide images (45). This model considers histopathological features from the tumor microenvironment. in gigapixel-sized WSIs in a semi-supervised manner and was trained to provide interpretable prognostic biomarkers in patients with kidney, breast, lung, and uterine cancers.

Treatment: The outcomes of cancer treatment are affected by several key factors, such as the patient`s health status, cancer subtype, and stage. Additionally, molecular cancer research has recently revealed the contribution of genetic mutations to patients` responses to a specific treatment. The complex interplay of the above factors in the real world poses a significant challenge for oncologists in selecting the appropriate treatment regimen for a specific patient.

In this scenario, AI is inherently a powerful approach to the integration and aggregation of intricate and multi-dimensional datasets and providing comprehensive data support for decision-making (46, 47). For example, Luo et al. proposed a collaborative filtering method with machine learning. It can identify the most suitable compounds for patients without genetic data, making it feasible to predict drug sensitivity and achieve personalized drug selection in a cost-effective way (48). Abajian et al. used Supervised Machine Learning with both Logistic Regression (LR) and Random Forest (RF) algorithms to explore the treatment response to transarterial chemoembolization for hepatocellular carcinoma. Both LR and RF models achieved an overall accuracy of 78% and identified cirrhosis status and relative tumor signal intensity (>27.0) as the two strongest predictors of treatment response (49). Kong et al. introduced a NetBio-based machine learning, which accurately predicted the treatment responses to Immune checkpoint inhibitors (ICIs) in three different cancer types-melanoma, gastric cancer, and bladder cancer. This model demonstrated superior performance in comparison with conventional ICI treatment biomarkers, such as the expression profiles of ICI targets (50).

Prognosis and management: Understanding the progression and survival time of patients is essential for cancer management. Oncologists used to predict patients’ prognoses based on their experience of understanding patients’ clinical profiles (age, health status) and tumor characteristics (subtype, stage, and grade). Nevertheless, this strategy is inherently limited and has a low predictive capability due to individual variation. AI has exhibited great promise to deal with these constraints and achieves accurate prognosis prediction for individual patients (51). Qiu et al. developed an XGBoost model to help physicians make clinical decisions. It employed clinicopathological information and predicted the risk of distant metastasis in patients with rectal cancer (52). Based on LASSO regression and Pearson correlation coefficients, Cai et al. identified metastasis-associated genes from different cancer tissues and then used them to build a CNN-based model, “Multi-Dimensional Convolutional Neural Network (MDCNN).” It achieved satisfactory prediction accuracy in bone metastasis, lung metastasis, and liver metastasis (53). The combination of AI and the Internet of Things (IoT) technology enables telemedicine and intelligent monitoring functions, allowing patients to receive scenario-based remote management (54).

Drug Discovery: Drug discovery and development of anti-cancer drugs is the goal of translational medicine. However, this work is quite a costly and time-consuming operation. Additionally, although the advances in muti-omics and clinical trials provide quite meaningful information, their complexity also imposes a huge obstacle. AI and computer-aided drug design, along with modern experimental technical knowledge, has energized data mining for faster drug design and development in the pharmaceutical industry (55). For example, AlphaFold2, a deep neural network algorithm, demonstrates high accuracy in predicting the three-dimensional structures of proteins, particularly when sequences of multiple homologs are available. It helps us understand protein function changes underlying carcinogenesis and improve our approaches to counter them (56). Meanwhile, low-cost cancer drug repurposing can be achieved by deep learning approaches, which aid the modeling of existing drugs for discovering novel drug targets. For example, Zhou et al. designed a prediction approach called an ensemble of multiple drug repositioning approaches (EMUDRA). Using EMUDRA, they predicted and experimentally validated the antibiotic rifabutin as an anti-cancer drug for triple-negative breast cancer (57).

## 3 Liver immune microenvironment and its alterations in cancer

The liver is not only an important metabolic organ but also possesses significant immune functions, and it contains a vast array of immune cells (**Table 1**). Several factors contribute to the unique immune functions of the liver. Firstly, the liver is a hematopoietic organ during embryonic development. Secondly, the flow of portal venous blood carries components from the gastrointestinal tract and spleen (58). Thirdly, the liver participates in mucosal immunity through the biliary system. Due to the liver’s direct exposure to many antigens from the gastrointestinal tract, it has developed a unique immune tolerance, which is manifested as intrinsic tolerance mechanisms in both innate and adaptive immune responses. Therefore, the liver can protect itself from autoimmune damage caused by the extensive presentation of gastrointestinal antigens (59, 60). However, in the context of liver injury and disease, various liver cells participate in complex pro-inflammatory responses, which may lead to hepatocyte death and further disease progression (61).

**Table 1:** Properties of immune cells in the liver.

Cell type	Markers	Functions in liver
Macrophages	F4/80, CD68 (Kupffer) CD86 (M1) CD68, CD163 (M2)	Engulf pathogens and dead cells, participate in antigen presentation, and produce various cytokines to regulate immune responses.
NK Cells	CD56, CD16	Identify and kill cells infected with viruses and tumor cells.
Dendritic Cells	CD1a, CD11c	Maintain immune tolerance and regulate liver-specific immune responses
cDCs	XCR1, CLEC9A (cDC1) CD11b, CD172a (cDC2)	Process antigens and present them to T cells, triggering an immune response against pathogens or tumor cells (cDC1). Induce regulatory T cell responses, promote coordination between humoral and cellular immunity. Regulate immune responses to pathogens in the liver and stimulate B cells to produce antibodies (cDC2).
pDCs	B220, PDCA-1 (mouse) BDCA-2, BDCA-4 (human)	When viruses invade, pDCs rapidly activate and secrete cytokines such as interferon, activating other immune cells and initiating an antiviral immune response.
Neutrophils	CD66b, Ly6G	The first line of defense in acute inflammation, phagocytosis and killing of invading microorganisms.
T Cells	CD3, CD4, CD8	Directly kill target cells or secrete cytokines to clear viral infections, monitor tumor development, and participate in liver transplant rejection responses.
CD4^+^ T	IFN-γ (Th1) IL-4, IL-5, IL-13 (Th2) IL-17 (Th17) CD4	Assist in immune response (activate other immune cells, promote antibody production), immune regulation (maintain immune balance, inhibit inflammatory response), and participate in liver repair (promote liver cell regeneration, regulate fibrosis).
CD8^+^ T	CD8	Have cytotoxic function, participates in adaptive immune responses, and can recognize and eliminate cells that are infected with viruses or have mutations.
NKT	CD161, NK1.1	Secrete cytokines to regulate immune responses, with anti-tumor and immune surveillance functions.
Tregs	CD4, CD25, FoxP3	Maintain liver immune tolerance.
B Cells	CD19, CD20	Produce specific antibodies, participate in antigen presentation, and regulate the activity of other immune cells.

Innate immune system: In the liver, the innate immune system forms the first line of defense against pathogens, present at birth and lasts throughout life. Immunity against various pathogens or malignant cells is provided by different types of immune cells (62). These cells include neutrophils, natural killer cells, Kupffer cells, monocytes, dendritic cells, and natural killer T cells (NKT) (63). Neutrophils, the most abundant group of circulating white blood cells, constitute the first line of defense in acute inflammation by phagocytosing and killing invading microorganisms (64). Natural killer cells Identify and kill cells infected with viruses and tumor cells (65). They don’t require secondary activation for their cytolytic activity. Instead, they induce apoptosis in tumor cells by activating FasL or TRAIL (66, 67). In case of their inactivation or restricted infiltration to the liver, tumor cells grow rapidly (68, 69). Kupffer cells are resident macrophages in the liver and constantly in contact with antigens from the gastrointestinal tract (70–72). Bleriot et al. identified two distinct populations of Kupffer cells, which share core molecular characteristics but express different genes and proteins (73). Additionally, the liver also recruits a large number of monocytes from peripheral blood and converts them into macrophages in the liver microenvironment (monocyte-derived macrophages). Different subtypes of macrophages can be distinguished with the specific expression of cell markers, such as CD11b, CCR2, and F4/80 (74–76). M1 macrophages mainly express CD16 and CD32, etc., and also produce TNFα, nitric oxide (NO), and reactive oxygen intermediates (ROI) to play antitumor roles, while M2 macrophages express several surface molecules such as CD163, Dectin-1, etc., and release interleukins (IL-4 and IL-13) and glucocorticoids, mainly perform the immunosuppressive pro-tumor activity (77, 78). Dendritic cells, also known as antigen-presenting cells, identify affected cells or pathogens and present them to other immune cells, thus maintaining immune tolerance and regulating liver-specific immune responses (79). NKT cells are unconventional T cells that are activated by glycolipid antigens (80, 81). They have both NK cell surface markers and antigen receptor characteristics of T cells and serve as a bridge between innate and adaptive immunity (82). The NKT cells that are located in hepatic sinusoids provide intravascular immune surveillance (83) where they may mediate proinflammatory effects through type I NKT cell subsets or exhibit immunosuppressive functions via type II NKT cells (84). In short, these immune cells coordinate with each other to accomplish the innate immune response in three steps: early inflammation, amplification of the inflammatory signal, and resolution.

Adaptive immune system: Several subtypes of T cells abundantly exist in healthy liver, including CD4^+^ helper T (Th) cells, CD8^+^ cytotoxic T cells, and regulatory T cells (Tregs) (85). CD4^+^ T cells are crucial for preventing tumorigenesis by facilitating the elimination of malignant cells (86–88). They typically act as initiators of antitumor responses and correlate with favorable responses to immunotherapy. CD8^+^ cytotoxic T cells serve as the primary effector cells of the cellular immune system, which recognize presented antigens and kill infected or malignant cells (89). Additionally, a population of CD8+ tissue-resident memory (TRM) cells exist in the liver, functioning as local immune sentinels (90, 91). Tregs are a subset of CD4^+^ T cells with immunosuppressive properties. These cells are crucial for maintaining homeostasis and immune tolerance (92, 93). Accumulation of Tregs has been implicated in facilitating immune evasion and hepatocarcinogenesis (92, 93).

B cells are a group of specialized cells that produce specific antibodies, participate in antigen presentation, and regulate the activities of other immune cells (94).

The development of liver cancer is highly related to infection and inflammation, which foster the unique Immunosuppressive microenvironment of liver cancer. It is characterized by blunted anti-tumor immunity, an enrichment of tumor-promoting immunosuppressive cell types, and impaired innate and adaptive immunity (95–99). Recently, immune checkpoint inhibitors (ICIs) have demonstrated promising clinical benefits in HCC, thus emphasizing the importance of immunotherapy (100). Apart from ICIs, immunotherapy also encompasses adoptive cell therapy, oncolytic virotherapy, and cancer vaccine therapy. These approaches can improve T-cell function and enhance cellular immunity, thereby leading to the elimination of LC-IME and the inhibition of tumor growth. To guide the application of immunotherapy, more efforts are needed to gain a deeper understanding of LC-IME.

## 4 The integration of AI and the immune microenvironment of liver cancer

In liver cancer, the intricate heterogeneity, consisting of diverse immune and stromal cells, significantly contributes to metastasis, relapse, and drug resistance (101–103). The exploration of the tumor immune microenvironment and complex cellular interactions can provide crucial insights for developing more effective, tailored immune-oncology therapies. However, the sheer volume and complexity of data from single-cell RNA sequencing (scRNA-seq) and multi-omics pose challenges for direct clinical application. In addressing these challenges, artificial intelligence (AI) is increasingly recognized as a potent tool that enhances our understanding of these large-scale datasets.

### 4.1 The integration of AI and omics data

scRNA-seq analysis: scRNA-seq allows researchers to conduct in-depth analysis of molecular characteristics, such as gene expression and epigenetic modifications within individual cells, generating vast amounts of genetic information data (104). The analysis of these data is crucial for revealing cellular heterogeneity and functional characteristics (105). With the intervention of AI, rapid processing and interpretation of massive scRNA-seq data can be achieved with enhanced data accuracy. AI algorithms can automatically identify and filter out noise, retaining the true biological differences between cells and thereby improving data reliability (106). Additionally, batch effects, a common issue in scRNA-seq analysis, can be caused by various factors such as experimental samples, platforms, and library construction methods. AI technologies can effectively eliminate these batch effects while preserving biological differences by projecting high-dimensional data into a low-dimensional cellular embedding space through an asymmetric autoencoder structure (107). In fact, these methods not only improve the accuracy of data integration but also enable online data integration and comparative analysis of new data with existing data.

By use of different machine learning approaches, cell type identification models are developed to recognize cell types and subtypes (108). These models can extract key biological insights to predict the changes in gene expression levels or even dynamic changes in gene interaction networks. For example, scRobust, a self-supervised learning strategy built on the transformer architecture, has demonstrated effectiveness in cell-type annotation and drug tolerance detection (109). A deep learning model “Enformer Celltyping” predicts epigenetic signals across cell types. It overcomes the limitations of existing machine learning approaches, which are confined to the cell types they were trained on (110). Here, we summarized cell-type identification models in **Table 2**.

**Table 2:** Cell-type identification models for scRNA-seq analysis.

Models	Paradigm	Algorithm
Scmap	Unsupervised-Graph based	Nearest neighbor
Seurat	Unsupervised-Graph based	Nearest neighbor
scType	Unsupervised-Graph based	Nearest neighbor
ScScope	Unsupervised-Deep learning based	Recurrent network
DESC	Unsupervised-Deep learning based	Autoencoder
ScAIDE	Unsupervised-Deep learning based	Autoencoder
scETM	Unsupervised-Deep learning based	Autoencoder
scVI	Unsupervised-Deep learning based	Hierarchical Bayesian
DISC	Semi-supervised-Deep learning based	Autoencoder
ScDCC	Semi-supervised-Deep learning based	Autoencoder
ScLearn	Supervised-Similarity-based	
CaSTLe	Supervised-General classifier-based	XGBoost
SCCAF	Supervised-General classifier-based	Logistic regression
ScID	Supervised-General classifier-based	Fisher’s linear discriminant analysis
ScDeepSort	Supervised-Deep learning based	Weighted GNN
NeuCA	Supervised-Deep learning based	Hierarchical FFNN
ItClust	Supervised-Transfer learning based	
SCTL	Supervised-Transfer learning based	

Multi-omics analysis: Complex and dynamic networks of molecules in LC-IME make a single layer of “omics” unable to provide deep insights into the underlying mechanisms. Recent technological advancement in high-throughput measurement of genome (111, 112), epigenome (113, 114), metabolome (115), transcriptome (116), and proteome (117) allows comprehensive multi-omic studies. Multi-omics approaches are pivotal in identifying new therapeutic targets (118) and predicting patients’ responses to treatments (119). The data from different omics can be cross-fused and mutually verified, providing a more reliable, comprehensive, and systematic perspective (120, 121). Through the integrated analysis of omics data, in-depth biological data that cannot be obtained by a single omics technology can be uncovered (122, 123). However, the advantage of multi-omics data integration comes with the extra complexity deriving from inherently diverse types of omics datasets, which may pose a challenge to integrateing the omics data in a biologically meaningful manner (124). The experimental data generated across diverse laboratories often cannot be seamlessly amalgamated due to inherent constraints. Additionally, the inherent heterogeneity of multi-omic datasets, stemming from technical, biological, chemical, and physical sources, poses significant challenges for interpretation (125).

With the continuous development of AI technologies, the integration of AI and multi-omics has emerged as one powerful solution to these challenges (126–128). AI has remarkable capabilities in deciphering complex patterns and extracting meaningful insights from large and intricate datasets (129–131). This enables researchers to more systematically analyze the complexity of biological systems (132), reveal the interactions and regulatory mechanisms between different molecular layers, and more accurately identify disease-related molecular markers and potential drug targets (133, 134). This subsequently contributes to the development of personalized medicine and precise treatment plans, improving therapeutic effects and reducing side effects (16, 135, 136).

Data-based Integration. This methodology has proven effective in several studies. Zhang Team merged information from single-nucleotide polymorphisms (SNPs) and transcriptomic profiles into a single matrix, which uses a Bayesian integrative model to facilitate the investigation of their interplay and enable the prediction of quantitative phenotypes (137). To predict remission rates and survival outcomes in ovarian cancer, Mankoo and colleagues integrated the data of copy number alteration, DNA methylation, microRNA, and gene expression and performed a multivariate Cox-LASSO analysis (138). Shen et al. proposed the iCluster framework for glioblastoma subtyping. This framework harmoniously and integrated, with a common set of latent variables, three distinct omics data of copy number alteration, gene expression, and DNA methylation (139).

Model-based Integration. In a model-centric integration framework, distinct models tailored to individual data perspectives are initially formulated, subsequently converging through a fusion process of their respective outputs. For example, the ATHENA tool (140–142), which is designed for investigating heritable and environmental network associations, integrates different omics data of copy number alterations, DNA methylation, miRNA, and gene expression to uncover correlations with clinical endpoints. This integration involves constructing foundational models and neural networks per omics type, ultimately leading to the construction of an integrated model (137). Wang’s team used Similarity Network Fusion (SNF) for cancer subtyping. It begins by creating patient similarity matrices based on DNA methylation and the expression of mRNA expression or miRNA and moves to an iterative nonlinear integration, where the three foundational similarity matrices converge into a unified matrix (143). To predict drug resistance in HIV protease mutants, Dr. Ghici and Potter devised an ensemble-based strategy. It sets up the basic predictive models with structural characteristics of the HIV protease-drug inhibitor complex and DNA sequence variations, respectively, and then orchestrates a majority voting system to enhance the accuracy of drug resistance prediction (144).

### 4.2 Current achievements of AI-guided scRNA-seq for cellular identification in LC-IME

#### 4.2.1 Neutrophil

Neutrophils play a key role during the initiation of innate immunity and the shaping of adaptive immunity. Several subtypes of Tumor-associated neutrophils (TANs) exist with different functions and markers: the antitumor N1, the protumor N2, and polymorphonuclear myeloid-derived suppressor cells (PMN-MDSCs) (145, 146). Tumor cells or other stromal cells in LC-IME educate TANs polarization towards pro-tumor phenotype through the secretion of cytokines or chemokines, such as GM-CSF, IL-6, TGF-β, and E2 PGE2 (147). Furthermore, the elevated neutrophil-lymphocyte ratio is associated with advanced cancer stage, aggressive tumor characteristics, as well as recurrence after resection but varies with etiology (148). Neutrophil extracellular traps (NETs), a unique structure produced during neutrophil death, have been shown to promote HCC metastasis by provoking inflammatory responses (149–152). However, due to the existence of high heterogeneity, some subsets of neutrophils have pro-tumor effects, while others appear to have anti-tumor effects, the overall influence of neutrophils on cancer therapy remains obscure (153–155). Zhang's team performed scRNA-seq analysis and stratified patients into five subtypes, including immune activation, immune suppression mediated by myeloid or stromal cells, immune exclusion, and immune residence phenotypes, which were spatially organized and associated with chemokine networks and genomic features. Notably, the abundance of tumor-associated neutrophils (TANs), particularly prominent within the myeloid-cell-dominated subtype, emerged as a harbinger of an adverse clinical prognosis. Depletion of TANs in mouse models significantly attenuated tumor progression, thereby shedding a promising light on therapeutic targets for innovative immunotherapeutic strategies (156). Neutrophils also showed resistance to anti-PDL-1 therapy in HCC via T-cell exhaustion (156). Interestingly, due to shorter lifespan and less abundance of RNAs, Neutrophils are difficult to identify by single-cell sequencing. However, application of optimized workflows (such as no enrichment strategy) (156) or capture methods (such as the BD Rhapsody platform) (157) made it possible to identify them. Still, some neutrophils with unique transcriptomic and functional features are identified in HCC by scRNA sequencing. Neutrophils expressing MMP8, CD74, SPP1, etc in HCC are considered tumor-associated neutrophils. Importantly, Particularly, CD10+ ALPL+ neutrophils hinder anti-PD-1 therapy by permanently destroying the T-cell (153). Suggesting that identifying and targeting neutrophils in HCC is essential for successful clinical outcomes.

#### 4.2.2 Macrophages

Tumor-associated macrophages (TAMs) are one of the most abundant innate immune cells and are observed at all stages of tumor progression in the LC-IME (95). According to the difference of functions in tumor progression, there are the classical M1 subtype and the alternative M2 subtype. The M1 phenotype is induced by pro-inflammatory cytokines such as IL-1β, IL-6, IL-12, and tumor necrosis factor-α (TNF-α), whereas the M2 phenotype is polarized by immunomodulatory molecules such as IL-4, IL-10, macrophage colony-stimulating factor (M-CSF), and transforming growth factor-β (TGF-β) (158). TAMs promote liver cancer progression through various approaches, including angiogenesis, cancer cell proliferation, immunosuppression, extracellular matrix remodeling, and drug resistance to therapeutic agents (159). TAMs express inhibitory immune checkpoint proteins, such as PD-1, PD-L1, and TIM-3, secret the immunosuppressive cytokine IL-6, and recruit Tregs (160–162). Furthermore, TAMs are important bridges between tumor cells and other immune effector cells. M2 TAMs secrete IGF-1 and CCL20 to recruit Tregs and impair CD8+ T cell function (163). FasL^+^CD11b^+^F4/80^+^ monocyte-derived macrophages interact with the activated antigen-specific Fas^+^CD8^+^ T cells and make them undergo apoptosis. The elimination of these hepatic macrophages significantly increased the survival of hepatic T cells (164). Moreover, Osteopontin (OPN), a pro-metastatic gene, promotes macrophage infiltration and PD-L1 expression in HCC by activating CSF1/CSF1R pathway (165). Conversely, upon appropriate stimulation, macrophages exhibit remarkable anti-tumor capabilities, such as phagocytosis of cancer cells and cytotoxic tumor eradication (159). Therefore, Macrophage-targeting strategies have the potential to synergize with current therapeutic tools to improve the outcomes of patients with liver cancer. Single-cell sequencing has identified several new subtypes of macrophages in HCC. For instance, there are two major types of macrophages: C1QA+ and THBS1+ macrophages (166). Among them, THBS1+ macrophages are myeloid-derived suppressor cells (MDSC)-like cells. However, C1QA+ are considered TAM-like macrophages, which have properties of both M1 and M2 macrophages and highly express APOE, GPNMB, and SLC40A1 (98) and are associated with poor prognosis of liver cancer. The accumulation of LAIR1+ and TIM3+ TAM macrophages reduced the infiltration of CD8+T cells and was associated with poor prognosis of HCC patients (167). Similarly, the abundance of the APOC1+ macrophages was comparatively higher in HCC tissues, inhibiting APOC1 improves the effects of anti-PD-1 therapy by reshaping M2 macrophages into the M1 macrophages (168). Considering the key role of macrophages (TAM) in cancer development, chemical inhibitors are being trialed, such as the combination of CCR2/CCR5 antagonists (targeting macrophages) with Nivolumab is currently in phase II clinical trials (NCT04123379). The complex functions of TAMs have sparked great interest in developing new therapeutic strategies targeting macrophages.

#### 4.2.3 Dendritic cells

Dendritic cells (DCs), functioning as antigen-presenting cells (APCs), interact with diverse immune cells and form a vital mediator between innate and adaptive immunity. There are two types of DCs, including Conventional DCs (cDCs) and plasmacytoid DCs (pDCs). The primary responsibility of cDCs (either cDC1 or cDC2) is antigen presentation, whereas pDCs are specialized for antiviral and antitumor immunity via the secretion of type I interferons (169),

In immunosuppressive tumor microenvironment, DC cells can be functionally reshaped and lose their antitumor functions. Tregs suppress the expression of HLA-DR and impair the antigen-presenting function of cDC2 cells (170, 171). DC cells often play an immunosuppressive role, and the enrichment of tumor-infiltrating pDCs was correlated with Tregs infiltration as well as poor prognosis in patients with HCC (172, 173). As the immune response to immunotherapy largely depends on DC cells, many strategies have been evaluated for stimulating DC cells in HCC patients, such as DC vaccines (174), nanodrugs (175, 176), and DC-derived exosomes (177), some of which have been demonstrated to activate tumor-specific immunity. Advanced scRNA-seq has identified the heterogeneous nature of dendritic cells (DC) in HCC, thus revealing diversity in their functions. These heterogeneous mature DCs, including CCR7+ LAMP3+ DCs, can migrate from tumors to lymph nodes, interfering with T cell function, including exhausted T cells (TEX) and Tregs cells (98). They are also found in lung cancer because they also express immune regulatory markers (Cd274, Pdcd1lg2, and Cd200) and maturation markers (Cd40, Ccr7, and Il12b); thus, they are named as mature dendritic cells enriched in immune regulatory molecules (mregDCs) (178). In the context of ICI treatment, a cellular triad composed of mregDCs, CXCL13+ helper T (Th) cells, and PD-1^hi^ progenitor CD8+ T cells is significantly enriched in the HCC microenvironment. Communication between mregDCs and CXCL13+ Th cells within these cellular triads helps in the differentiation of progenitor CD8+ T cells into effector antitumor CD8+ T cells (179). Similarly, CXCR3+ CD8+ effector memory T (TEM) cells and HLA-DR+ cDC1 recruited to determine the responsiveness of HCC to ICI (180).

#### 4.2.4 T cells

CD8+T cells exhibit an exhausted phenotype and are incapable of halting tumor progression in HCC, and the enrichment of exhausted CD8+T is negatively correlated with the response to immunotherapy and prognosis in patients in patients with HCC (181). It has been demonstrated that dysfunction of CD8^+^ T cells occurs within a few hours after they encounter tumor antigens, even before undergoing cell division T (182). This rapid divergence of T cell fate prior to cell division provides us a clue for timely application of immunotherapy. Additionally, tissue-resident memory CD8+ T (TRM) cells are also enriched in tumors, especially in HBV-related HCC (183). A high TRM proportion is associated with better outcomes following ICI therapy (181, 184).

Under the co-stimulation of activated APCs and different cytokines, Naïve CD4+ T cells proliferate and differentiate into different subsets, including Th cells (specifically, Th1, Th2, and Th17), follicular helper T (Tfh) cells, and Treg cells (185). Among them, Th1 cells secret IFN-γ and IL-2 and promote the anti-tumor effect of CD8+ T cells (186). Furthermore, Th1 cells facilitate dendritic cell (DC) maturation through the CD40-CD40L signaling axis (187). On the other hand, Th17 cells are abundant in HCC and are associated with unfavorable clinical outcomes (188). Moreover, Th17 cells contribute to resistance against PD-L1 therapy by upregulating PD-L1 expression in HCC cells through the secretion of IL-17A (189). Treg cells are significantly increased in HCC and are correlated with dysfunction of CD8 T-cells, reduced clinical benefits of anti-PD-L1 plus anti-VEGFR, and poor survival (190–192). Given the crucial role of Tregs in maintaining immune homeostasis and preventing auto-immune diseases, there is a pressing need for innovative approaches that precisely target tumor-infiltrating Tregs and spare the physiological function of Tregs. Currently, little is known about the roles of Th2 and Tfh cells in HCC, which require further investigation. Both exhausted CD8+T and Treg cells are characterized by upregulated expression of a series of inhibitory receptors, such as PD-1 and CTLA-4 (193, 194). In pre-tumoral HCC tissues, monocytes express higher levels of CD93, which inhibit the infiltration of CD8 T cells (195). Thus, targeting CD93-expressing monocytes can help increase the activation and infiltration of the CD8 T cells. Multi-omics is commonly used to identify the T-cells in tumors, but standard AI-based systems for efficiently detecting T-cells in liver TME are not available. Questions regarding advanced AI intervention in the detection of T cells in solid tumors, especially liver cancer, remain unclear.

#### 4.2.5 B cells

Tumor-infiltrating B cells play pivotal roles in tumor immunity, including antigen presentation, antibody production, and other functions (196). Their presence is notably a favorable marker for HCC prognosis (94, 197). Additionally, the presence of intra-tumoral tertiary lymphoid structures (TLS) is correlated with a reduced risk of early recurrence in HCC patients after surgical resection (198). Within TLS, abundant B cells transform into plasma cells and produce IgG antibodies that effectively combat tumors (199). However, there are some subtypes of B cells that play pro-tumor roles in HCC. IgA-producing B cells enhanced the expression of PD-L1 and exert an inhibitory influence on T-cell activation (200). Furthermore, regulatory B cells (Bregs), which are characterized by IL-10 secretion, not only dampen T-cell responsiveness but also contribute to HCC progression via the CD40/CD154 signaling axis (201). Given the intricate and diverse functions exhibited by various B-cell subsets in HCC, further research endeavors are required to unlock their full potential in therapeutic strategies. As aforementioned, single-cell sequencing is commonly used to identify the cellular composition of tumors. The level of B cells in liver tumors was detected using conventional sc-seq (202), but AI-guided sc-seq is not generally applied.

#### 4.2.6 NK cells and other innate lymphoid cells

Innate lymphoid cells (ILCs) are a highly heterogeneous family, which comprise NK cells, ILCs also include ILC1s, ILC2s, and ILC3s. In the human liver, NK cells constitute a prominent subtype of lymphocyte, accounting for about 50% of the total intrahepatic lymphocytes (203). These NK cells can be categorically split into two distinct subsets: cytotoxic NK cells marked by CD56^dim^CD16^high^ expression and immunoregulatory NK cells characterized by CD56^bright^CD16^low^ expression (204). Cytokines, such as IL-10 and TGF-β, induce the exhaustion phenotype of CD11b-/CD27-NK cells through the upregulation of NKG2A and CD96, respectively. The blockade of IL-10 or TGF-β pathway can reverse the dysfunction of NK cells (205, 206). Furthermore, a significant reduction of NAD+ in NK cells causes their dysfunction. Supplementation with NMN, a NAD+ precursor, restores the anti-tumor effects of NK cells (207). Due to its potent cytotoxicity against tumors without dependence on secondary activation, various innovative NK cell-based therapeutic strategies have been explored in HCC (208). For example, bispecific antibodies are designed to bridge the gap between NK cells and tumor cells (209–211). These antibodies possess dual specificity, enabling them to simultaneously bind to a tumor-associated antigen on HCC cells and an activating receptor on NK cells. This interaction triggers potent antibody-dependent cellular cytotoxicity (ADCC), NK cells are activated and directed toward the tumor, leading to their direct killing via the release of cytotoxic granules containing perforin and granzymes. Furthermore, adoptive cell transfer (ACT) therapies have emerged as a promising avenue for NK cell-based treatments. NK cells are either expanded ex vivo or subsequently modified to enhance their tumor-targeting and killing capabilities. These modifications can be achieved through the engineering of chimeric antigen receptors (CARs) into NK cells (212) or the activating killing-ability of NK cells with cytokines (213).

Not like the cytolytic NK cells, ILC1s, ILC2s, and ILC3s function through cytokine secretion. Interestingly, the secretion behaviors of ILC1s, ILC2s, and ILC3s mirror the functions and characteristics of CD4+ helper T cell subsets. The three ILC subtypes secrete IFN-γ/TNF-α, IL-4/IL-5/IL-13, and IL-17/IL-22, which are signatures of characteristics of Th1, Th2, and Th17 cells, respectively (204, 214). Currently, their roles in liver cancer are still controversial. For example, ICOS+ILC2a cells were enriched in HCC and associated with poor prognosis (215). However, in another study, a high ILC2/ILC1 ratio is associated with enhanced anti-tumor immune responses and better prognosis (216). Further studies are needed to define the contribution of these cytokine-secretion ILCs in HCC.

## 5 Challenges and Future Prospects

In the past several years, a drastic rise in data digitization has been seen in many sectors, including the medical sector. However, it comes with challenges, especially in acquiring and scrutinizing suitable data to solve various complex problems. LC-IME is highly heterogeneous and complex, and so far, no AI system has been constructed to identify various cell types and signaling pathways (217, 218). The cellular composition of the LC-IME is usually determined by conventional single-cell sequencing; AI intervention can improve the overall outcome. With enhanced automation, AI has the power to handle large-scale data because AI-guided tools can learn from input data and independently draw conclusions according to the given objectives. On the other hand, conventional methods require stepwise monitoring and human input for manual analysis and drawing conclusions. However, several limitations still exist for freely applying AI in LC-IME. First, there is a long way to go in building highly accurate AI algorithms and models that are both explainable and trustworthy. Further integration of constraints into the models, based on biological domain knowledge in a principled manner, is necessary to improve both the accuracy and interpretability of models being applied to LC-IME. Second, the reliability of most studies is limited by small sample sizes. Future rigorous, large-scale longitudinal studies on LC-IME are needed for feature decomposition and to reduce the large number of variables. Third, research growth in this area is hindered by the lack of international guidelines or models that specify where AI is more likely to be useful in monitoring the integration of large data. There is a need for transparent, accessible, and curated data sharing. Moreover, interdisciplinary approaches, supplemented by rigorous co-production and co-design processes alongside individuals with liver cancer, are key to progress in this area. These research directions are expected to drive the in-depth application of AI technology in LC-IME field, thereby providing new solutions for precision medicine of liver cancer and significantly improving treatment outcomes and quality of life for patients.

## 6 Conclusion

With the rapid development of high-throughput sequencing technology and computer science, the amount of large omics data has increased exponentially, the advantages of multi-omics analysis have gradually emerged, and the application of artificial intelligence has become more and more extensive. Overall, this review has highlighted the potential, current applications, and implementation framework for integrating AI in the discovery and validation of biomarkers in HCC. Finally, we briefly explained the current challenges of multi-omics analysis and artificial intelligence in order to provide new research ideas for the medical industry and to promote the development and application of precision medicine.
